# Unusual Presentation of Cryptogenic Organising Pneumonia as a Focal Lung Mass: A Case Report and Literature Review

**DOI:** 10.1002/rcr2.70138

**Published:** 2025-03-05

**Authors:** Boon Hau Ng, Hsueh Jing Low, Nik Nuratiqah Nik Abeed, Nor Safiqah Sharil, Rose Azzlinda Osman, Andrea Yu‐Lin Ban

**Affiliations:** ^1^ Respiratory Unit, Department of Medicine, Hospital Canselor Tuanku Muhriz, Faculty of Medicine Universiti Kebangsaan Malaysia Kuala Lumpur Malaysia; ^2^ Department of Anesthesiology and Critical Care, Hospital Canselor Tuanku Muhriz, Faculty of Medicine Universiti Kebangsaan Malaysia Kuala Lumpur Malaysia

**Keywords:** focal lung mass, organising pneumonia, steroids

## Abstract

Organising pneumonia (OP) is a distinct pathological pattern characterised by the presence of granulation tissue buds composed of fibroblasts and myofibroblasts embedded in a loose connective tissue matrix within the distal pulmonary airspaces. When OP occurs without an identifiable cause or etiologic context, it is termed cryptogenic organising pneumonia (COP). The diagnosis of OP can be challenging due to its diverse clinical presentations, including the idiopathic form and various secondary forms associated with underlying diseases. We report a case of a middle‐aged male presenting with intermittent cough and haemoptysis. Initial sputum analysis was unremarkable, and the patient showed no improvement with antibiotic therapy. Chest radiography showed left lower zone consolidation. Computed tomography (CT) thorax revealed a mass in the left lower lobe, while positron emission tomography‐computed tomography (PET/CT) demonstrated a hypermetabolic lesion at the same site. Bronchoscopic bronchoalveolar lavage was negative for tuberculosis, respiratory pathogens, and malignancy. Autoimmune screening yielded negative results. A transthoracic tru‐cut lung biopsy confirmed the diagnosis of OP. The patient was treated with prednisolone, leading to significant clinical improvement and complete resolution of the lesion on follow‐up CT imaging.

## Graphical abstract

The diagnosis of organising pneumonia (OP) can be challenging due to its diverse clinical presentations. We report the diagnostic work‐up and treatment for a case of a middle‐aged male presenting with intermittent cough and haemoptysis, eventually diagnosed with OP.

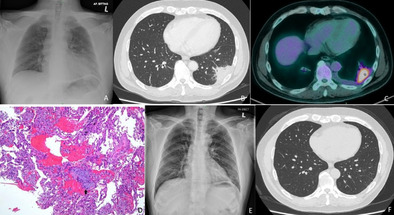

## 1 Introduction

Cryptogenic organising pneumonia (COP) is an idiopathic form of diffuse interstitial lung disease characterised by alveolar injury that triggers the formation of organised granulation tissue. This granulation tissue obstructs the alveoli and bronchioles, potentially progressing to respiratory failure. A definitive diagnosis of COP requires (1) a compatible clinical and radiological presentation, (2) identification of the characteristic pathological pattern on lung histopathology, and (3) exclusion of secondary causes. The incidence and prevalence of COP remain poorly defined, although it typically manifests in the fifth or sixth decade of life. The estimated annual incidence is approximately 1 per 100,000 individuals [1, 2].

## 2 Case Report

**FIGURE 1:**
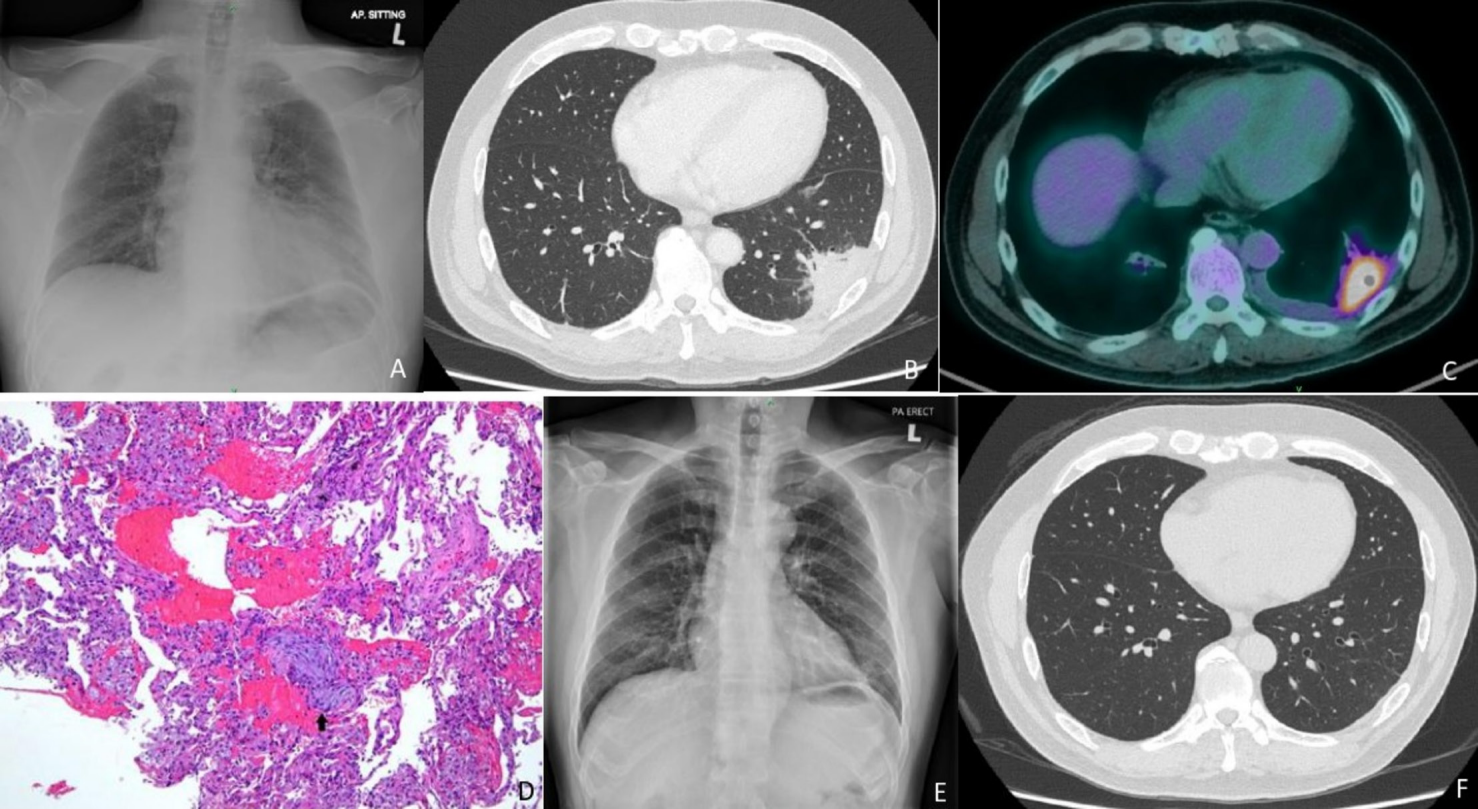
(A) Chest radiograph reveals consolidation in the left lower zone. (B) Contrast‐enhanced computed tomography showed a consolidative mass in the left lower lobe. (C) PET/CT scan demonstrates a hypermetabolic mass in the left lower lobe. (D) Fragments composed of benign pneumocytes lining the alveolar spaces, with scattered foamy macrophages, haemorrhage and occasional fibroblastic foci within the space (arrow). H&E, ×100. (E) A 6‐week follow‐up chest radiograph post‐prednisolone treatment showed improvement in the left lower zone consolidation. (F) Contrast‐enhanced CT at 3 months post‐prednisolone treatment indicates resolution of the left lower lobe consolidation.

A man in his mid‐50s presented to a tertiary hospital with a two‐week history of intermittent cough and haemoptysis. His medical history was significant for hypertension and dyslipidaemia. He was a non‐smoker. Initial sputum analysis for *Mycobacterium tuberculosis* and bacterial cultures were negative. Laboratory investigations revealed a C‐reactive protein (CRP) level of 38 mg/L, a white cell count (WCC) of 12 × 10^9^/L, and an erythrocyte sedimentation rate (ESR) of 108 mm/h. A chest radiograph demonstrated consolidation in the left lower zone (Figure 1A). Despite two courses of antibiotics, the patient's symptoms persisted. A computed tomography (CT) scan of the thorax showed a mass in the left lower lobe (Figure 1B), which was further evaluated using positron emission tomography‐computed tomography (PET/CT). The PET/CT scan revealed a hypermetabolic subpleural mass in the lateral basal segment of the left lower lobe, measuring 4.4 × 2.4 cm with a maximum standardised uptake value (SUVmax) of 10.7, and a mild pleural effusion was observed (Figure 1C).

An initial transthoracic needle biopsy was inconclusive. The patient declined a surgical lung biopsy and subsequently sought a second opinion at our centre. Upon presentation, he continued to experience intermittent haemoptysis and left‐sided chest pain. On examination, his vital signs included a blood pressure of 126/72 mmHg, a heart rate of 102 beats per minute, a temperature of 37.5°C, and an oxygen saturation of 94% on room air. Physical examination revealed no signs of connective tissue disease or cervical lymphadenopathy. Lung auscultation identified crepitations at the left lung base.

Repeat laboratory tests showed a WCC of 16 × 10^9^/L, haemoglobin of 13 g/dL, platelet count of 280 × 109^9^/L, CRP of 28 mg/L, and ESR of 120 mm/h. Chest radiography confirmed consolidation in the left lower zone. Bedside thoracic ultrasonography demonstrated a hypoechoic effusion occupying less than one rib space in the left lower zone, with an air bronchogram in the left lower lobe. Sputum analysis for tuberculosis, including acid‐fast bacilli (AFB) smear and *M. tuberculosis* polymerase chain reaction (PCR), was negative. Bacterial cultures showed no growth.

A follow‐up CT scan revealed an enlarging, enhancing consolidative mass in the left lower lobe, with associated pleural effusion, raising suspicion for malignancy. Bronchoscopy showed no endobronchial abnormalities, and bronchoalveolar lavage tests for AFB smear, *M. tuberculosis* GeneXpert, tuberculosis culture, respiratory pathogen PCR, and cytology were negative. Autoimmune screening, including antinuclear antibodies (ANA), extractable nuclear antigen (ENA) panel, perinuclear anti‐neutrophil cytoplasmic antibodies (pANCA), cytoplasmic ANCA (cANCA), and a myositis panel, was all negative. The patient again declined video‐assisted thoracoscopic surgery (VATS) for lung biopsy.

A repeat transthoracic tru‐cut needle biopsy, guided by PET/CT, targeted the hypermetabolic site was performed. Histopathological analysis confirmed OP (figure D). Pulmonary function tests were within normal limits: forced expiratory volume in one second/forced vital capacity (FEV_1_/FVC) ratio of 0.94, FEV_1_ of 2.96 L (86% predicted), FVC of 3.78 L (80% predicted), total lung capacity of 92% predicted, residual volume of 106% predicted, and diffusing capacity of the lungs for carbon monoxide of 96% predicted.

Based on clinical and histopathological findings, a diagnosis of COP was made. The patient began treatment with prednisolone at 0.5 mg/kg/day, starting with 35 mg once daily for 2 weeks, then gradually tapered over 6 months: 30 mg for 2 weeks, 25 mg for 4 weeks, 20 mg for 6 weeks, 10 mg for 6 weeks, and 5 mg for 6 weeks. His cough and haemoptysis resolved promptly after initiating corticosteroid therapy. Serial CRP levels showed a decreasing trend, and follow‐up chest radiographs demonstrated gradual resolution of the left lower zone consolidation (Figure 1E).

At the six‐week follow‐up, thoracic ultrasonography confirmed complete resolution of the pleural effusion. A CT scan at 3 months showed full resolution of the consolidative mass (Figure 1F). The patient completed a 6‐month course of prednisolone and remained well at the latest follow‐up, with no recurrence observed on serial chest radiographs.

## 3 Discussion

Organising pneumonia (OP) is a pulmonary repair process that can be triggered by various aetiologies, including infections, connective tissue diseases, and malignancies, or it may occur without an identifiable cause, termed cryptogenic organising pneumonia (COP). Histopathologically, OP is marked by the presence of fibrous tissue proliferation within the alveolar sacs and ducts, forming Masson bodies, which may extend into the bronchioles and obstruct the lumens [3]. COP typically develops subacutely, presenting with symptoms such as cough, fever, reduced exercise tolerance, and chest pain. Haemoptysis and pneumothorax are rare, with haemoptysis reported in less than 5% of cases [2]. As illustrated in our case, the unusual presentation of haemoptysis and a focal lung mass made the diagnosis challenging and required the exclusion of lung cancer through tissue biopsy.

Characteristic CT findings in OP include multiple alveolar opacities, solitary opacities, and infiltrative patterns. High‐resolution CT (HRCT) typically shows multifocal, fluctuating parenchymal consolidations that are often subpleural and peri‐bronchovascular, predominantly affecting the lower lobes in a bilateral and asymmetrical distribution [4]. Associated features may include ground‐glass opacities, air bronchograms, and traction bronchiectasis. A hallmark of OP is the migratory nature of the lesions, where some areas resolve spontaneously as new ones appear. OP can also present as solid, mixed‐density, or ground‐glass nodules with variable distributions, either scattered or peribronchovascular [5].

Bronchoscopy and BAL are used to exclude other potential causes of the observed lesions, such as infections, malignancies, eosinophilic pneumonia, and alveolar haemorrhage. BAL fluid often reveals a high lymphocyte percentage (20%–40%), with eosinophils and neutrophils accounting for 7%–10% [6]. Approximately 40% of patients show lymphocytosis with a decreased CD4+/CD8+ ratio, although this is not pathognomonic [6]. An increased eosinophil percentage may indicate overlap syndromes, such as OP with chronic eosinophilic pneumonia.

The diagnosis of COP relies on clinical and radiological assessment, exclusion of secondary causes, and histopathological confirmation via open lung biopsy or transbronchial lung biopsy [7]. VATS is preferred for obtaining adequate lung tissue to evaluate lesion distribution and lung architecture, facilitating a definitive diagnosis and ruling out other conditions. Although VATS is the gold standard for histopathological diagnosis, it is not always necessary; transbronchial biopsies may suffice in some cases, especially when characteristic granulation tissue is identified [8]. Core needle biopsy may be appropriate for focal consolidations, with the diagnosis reconsidered if there is a lack of response to corticosteroid therapy.

Corticosteroid therapy remains the cornerstone of COP treatment, often resulting in rapid clinical and radiological improvement, though relapses are common upon dose reduction or cessation. No controlled trials have defined the optimal treatment regimen or duration, and cases have been reported to vary widely in dosing and treatment length (Table 1). Consensus recommendations primarily guide current practices. The British Thoracic Society suggests starting prednisone at 0.75–1 mg/kg per day, tapering over 6–12 months [2, 9]. Alternatively, a three‐month course of 1–1.5 mg/kg of prednisolone with gradual tapering or initial methylprednisolone (0.5–1 g IV for 3 days) followed by prednisolone 20 mg daily may be used, adjusting based on clinical response. The Groupe d'Etudes et de Recherche sur les Maladies Orphelines Pulmonaires proposes a standardised protocol beginning with prednisone 0.75 mg/kg/day for 4 weeks, with a structured tapering schedule [2]. In severe cases, initial high‐dose IV methylprednisolone (2 mg/kg/day for 3–5 days) is recommended.

**TABLE 1:** A literature review on the reported cases of COP.

Author/year	Gender/age (years)	Presenting symptoms	Duration	Modality of biopsy	CT findings	Rx regime	Rx duration
Martin et al./2023 [10]	F/22	Fever, sore throat, dry cough, fatigue, nausea	6 weeks	TTNB	Basilar predominant GGO with central lucency	Steroids (1 mg/kg) and tapper	3 months
Vijayaravindh et al./2022 [11]	F/19	Cough, fever, weight loss	1 months	TBLB, SLB	Diffuse bilateral miliary nodules	Steroids	—
Lee et al./2022 [12]	F/35	SOB, cough, chest pain, fever	10 days	TBLB	Multifocal patchy airspace consolidation, GGO	Pred 0.5 mg/kg/d	—
Christian et al./2021 [13]	M/72	Cough	1 weeks	VATS	Consolidation, GGO	Pred, MTP, IVIg 0.4 g/kg/dose	8 weeks
Domas et al./2021 [14]	F/54	SOB	—	TBLB (cryobx)	Multiple bilateral confluent irregular foci, GGO, traction bronchiectasis	Pred 30 mg OD and tapper	6 months
Jakrin et al./2020 [15]	M/66	SOB, cough, weight loss, night sweats	2 weeks	TBLB, TTNB, VATS	Diffuse centrilobular micronodular infiltrations	MTP 1 g/d for 3d; pred 95 mg OD and tapper	—
Yao et al./2020 [16]	M/70	Cough, hemoptysis	1 month	TTNB	Peripheral consolidation	0.5 mg/kg/d and tapper	6 months
Huo et al./ 2018 [17]	M/61	Haemoptysis	—	SLR	Irregular mass with rough edges	SLR	—
Ailing et al./2017 [18]	M/65	Cough, chest pain	1 month	TTNB	Consolidation	0.75 mg/kg/d and tapper	6 months
Kang et al./2017 [19]	M/56	SOB	—	VATS	Pneumothorax, effusion, peripheral consolidation	—	—
	F/82	SOB, cough	5 days	—	Consolidation, GGO, pneumothorax	Pred 40 mg OD	—
Gaetano Rea et al./2016 [20]	F/57	SOB, cough, fever	4 months	No biopsy	Consolidation, mediastinal lymphadenopathy	Pred 0.50 mg/kg/d and tapper	3 months
Ding et al./2015 [21]	F/58	Cough, fever, fatigue	9 months	TTNB	Bilateral nodular, patchy alveolar opacities, thickened pleura	Pred 0.75 mg/kg/d	3 months

Abbreviations: COPD: chronic obstructive pulmonary disease; F: female; M: male; Pred: prednisolone; SLB: surgical lung biopsy; SLR: surgical lung resection; SOB: shortness of breath; TTNB: transthoracic needle biopsy; VATS: video‐assisted thoracoscopy.

Relapses occur in approximately 58% of cases, with multiple relapses in 19%. These are generally managed by increasing prednisone to 20 mg daily before tapering again. If relapse occurs while on a dose higher than 15–30 mg/day, a thorough reassessment of the diagnosis, including clinical, radiological, and pathological discussion, is warranted, especially if a large biopsy sample is not available.

Cryptogenic organising pneumonia remains a diagnostic and therapeutic challenge due to its nonspecific clinical and imaging features. Although corticosteroid therapy is effective in inducing remission, the risk of relapse requires individualised treatment strategies and long‐term monitoring. A thorough and systematic approach is essential in evaluating unresolved pneumonia to identify underlying causes, guide appropriate treatment, and prevent missed diagnoses.

## Author Contributions

B.H.N., H.J.L., N.N.N.A., and N.S.S. wrote up the case under the continuous supervision of A.Y.L.B. They discussed the case presentation, investigations, and patient management. R.A.O. was involved in the patient's management. B.H.N. and A.Y.L.B. supervised and guided the patient's management.

## Ethics Statement

The authors declare that appropriate written informed consent was obtained for the publication of this manuscript and accompanying images.

## Conflicts of Interest

Andrea Ban Yu‐Lin is an Editorial Board member of Respirology Case Reports and a co‐author of this article; she was excluded from all editorial decision‐making related to the acceptance of this article for publication.

## Data Availability

The data that support the findings of this study are available from the corresponding author upon reasonable request.
